# Impact of compensatory mechanism on health-related quality of life in adult spinal deformity surgery

**DOI:** 10.1186/1748-7161-10-S1-O54

**Published:** 2015-01-19

**Authors:** Sho Kobayashi, Tomohiko Hasegawa, Yu Yamato, Tatsuya Yasuda, Hideyuki Arima, Daisuke Togawa, Yukihiro Matsuyama

**Affiliations:** 1 Department of Orthopedic Surgery, Hamamatsu University School of Medicine, Japan

## Objectives

Global sagittal spinal imbalance leads to compensatory pelvic retroversion and femoral flexion that is far from economical standing posture. Recent reports showed spinal instrumentation surgery provided remarkable restoration of spinal alignment and better health-related quality of life (HRQOL) by adult spinal deformity surgery. However, there is a few previous reports about the impact of pelvic and lower extremity sagittal alignment on HRQOL by adult deformity surgery. Thus, we analyzed the effect of compensatory mechanism in adult spinal deformity surgery. The purpose of this study was to report radiological and clinical outcome with the patients' compensatory mechanism following adult deformity surgery.

## Materials and methods

This is a retrospective study at a single institute. A total of 60 patients who underwent posterior corrective thoracolumbar osteotomy were included in this study. Every patient were radiographically evaluated by sagittal vertical axis (SVA), Pelvic tilt (PT), Angle of Femoral Obliquity (FOA) on standing lateral radiographs, Oswestry Disability index (ODI) and Visual analog scale (VAS), SRS22 preoperatively and at 1 year after surgery. The subjects were classified into 4 groups according to SVA, PT and FOA: A. ideal balance group (normal SVA and PT). B. economical balance group (SVA>Femoral Head (FH) and normal PT). C. compensated balance group (SVA>FH, PT>22 and normal FOA). D. poor balance group (SVA>FH, PT>22 and FOA>10). Data were analyzed using t-tests and statistical significance was set at the p<0.05.

## Results

The patient population included 10 Males and 50 Females with a mean age of 69 years (range 37-84 year). Patients in group D (n=13, abnormal SVA, PT and FOA) retain the lowest HRQOL compared with the other patients preoperatively. The difference was statistically significant. At 1 year after surgery VAS, ODI and SRS22 were 2.5, 29 and 3.5 in group A (ideal, n=12). In group B (economy, n=13) they were 4.1, 39 and 3.1. In group C (compensated, n=32) they were 4.5, 40 and 3.1. In group D (poor, n=4) they were 5.8, 51 and 2.6. In group A and the other group (B, C, D) was statistically significant. Preoperative and Postoperative follow-up showed that HRQOL worsened among compensated balance patients.

## Conclusions

This is the first report that investigates the effect of compensatory mechanism on HRQOL in adult spinal deformity surgery. Our goal of adult spinal deformity surgery is not only to achieve good spinal balance but also not to compensate at standing posture.

